# Toward Deeper Unconscious Racial Bias Work in Education

**DOI:** 10.1177/01614681221142535

**Published:** 2022-11-29

**Authors:** Arlo Kempf

**Affiliations:** 1 Ontario Institute for Studies in Education, University of Toronto, Toronto, ON, Canada

**Keywords:** Unconscious racial bias, implicit racial bias, education, whiteness, white fragility, white supremacy, teachers, anti-racism, critical race theory, Ontario, Canada

## Abstract

**Background/Context:**

Unconscious racial bias (URB) can be a pernicious form of racism. In light of increased awareness of and research on the subject, URB work has become a key focus of equity work in health care, education, and corporate contexts as part of broader calls for racial justice. In Canada, targeting URB in education has become a policy priority at the national, provincial, and school board levels. The role of individual and organizational URB is now widely recognized in policy as central to equitable outcomes in schooling; however, research is limited on how to engage these forms of racism in educational contexts. Prevailing approaches to URB work in schools often include truncated one-off workshops, which leave unaddressed the connections between the individual racial biases, and the operations of white supremacy and racism at the institutional, systemic, and structural levels.

**Purpose/Objective/Research Question/Focus of Study:**

While URB is increasingly well-understood by social psychologists, there has been limited engagement from critical scholars working in areas such as critical race theory (CRT), anti-colonialism, and critical whiteness studies—despite the popularity of interrogating URB as an anti-racism strategy in education. CRT in education has laid bare and problematized the central function of schooling in the safeguarding and management of white supremacy. This project emerged from a dual recognition of URB as a productive entry point for racial awareness and anti-racism work, alongside a significant concern about the failure of mainstream URB discourse to address structural racism and white supremacy—masking at times the deeper ways that Euro-colonial racism underpins social relations in contemporary U.S., Canadian, European, and other contexts. This work seeks to address these limitations in the design of the study through deep work with participants. Specifically, the study sought to understand better the impacts of reading critical texts focusing on systemic, structural, and institutional racism on teachers’ understandings of their own racial biases, as well as teachers’ perspectives on the impacts of reading critical texts in terms of their professional practices.

**Research Design:**

This article reports on the findings of a 10-month study with secondary teachers in Toronto, Canada, focusing on critical approaches to racial bias mitigation in education. In addition to asking participants to enact a series of URB mitigation strategies developed in the field of social psychology, this study also required participants to read and reflect on one of the following critical anti-racism nonfiction texts: *White Fragility: Why It’s So Hard for White People to Talk About Racism* by Robin DiAngelo (2018); *Policing Black Lives: State Violence in Canada From Slavery to the Present* by Robyn Maynard (2017); *Everyday Antiracism: Getting Real About Race in School*, edited by Mica Pollock (2008); *Unsettling the Settler Within: Indian Residential Schools, Truth Telling, and Reconciliation in Canada* by Paulette Regan (2014); and *Monstrous Intimacies: Making Post-Slavery Subjects* by Christina Sharpe (2010). The project was designed using multiple data sources from participants, including electronic survey responses, ongoing journaling/reflection, a midpoint check-in questionnaire, and a final interview. These multiple entry points, as well as the duration of the project, aimed to go beyond the taken-for-granted and toward deeper understanding over time.

**Conclusions/Recommendations:**

Findings suggest that reading these works impacted teachers’ understandings of race and racism in terms of their teaching, as well as in terms of their personal relationships to race and racism, increasing their inclination and ability to address race and anti-racism. This work allowed for critical reflection to seep into the most intimate and invisible moments of operationalized whiteness in the professional and personal spheres of participants. This suggests an important complementarity between teacher intervention practices emerging from social psychology, and the introduction and engagement of critical anti-racist and anti-colonial texts in terms of teachers’ work for racial justice.

Unconscious bias, also known as implicit bias, can impact the way referees call a play (Parsons et al., 2011), the way orchestra leaders judge an audition (Goldin & Rouse, 2000), and the way teachers teach children (Starck et al., 2020). Unconscious racial bias (URB) can be a pernicious form of racism. In light of increased awareness and research on the subject, URB work has become a key focus of equity work in health care, education, and corporate contexts (Staats, 2016) as part of broader calls for racial justice. In Canada, targeting URB in education has become a policy priority at the national (Council of Ministers of Education of Canada, n.d.), provincial (Government of Ontario, 2017), and school board levels (Toronto District School Board, 2017). The role of individual and organizational URB is now widely recognized in policy as central to equitable outcomes in schooling; however, research is limited on how to engage these forms of racism in educational contexts.

This article reports on the findings of a 10-month study with secondary teachers in Toronto, Canada, focusing on critical approaches to racial bias mitigation in education. In addition to asking participants to enact a series of URB mitigation strategies developed in the field of social psychology, this study also required participants to read and reflect on one of the following critical anti-racism nonfiction texts: *White Fragility: Why It’s So Hard for White People to Talk About Racism* by Robin DiAngelo (2018); *Policing Black Lives: State Violence in Canada From Slavery to the Present* by Robyn Maynard (2017); *Everyday Antiracism: Getting Real About Race in School*, edited by Mica Pollock (2008); *Unsettling the Settler Within: Indian Residential Schools, Truth Telling, and Reconciliation in Canada* by Paulette Regan (2014); and *Monstrous Intimacies: Making Post-Slavery Subjects* by Christina Sharpe (2010); hereafter, these works are referred to collectively as the selected literature.

Braiding social psychology interventions with critical reflection approaches from education, the project significantly impacted most participants, who reported changes to thinking and behavior in and around racism and white supremacy^1^ in both the personal and professional realms of their lives. This article explores teacher reflections on the selected literature. Findings suggest that reading these works impacted teachers’ understandings of race and racism in terms of their teaching and in terms of their personal relationships to race and racism, increasing their inclination and ability to address race and anti-racism. Findings also suggest that reflexive and sustained engagement with critical anti-racism texts deepened participants’ learning and engagement with social psychology antibiasing techniques.

Given that I am a White, mainly hetero cis male who has spent most of his life in the United States and Canada, my biases are informed by these contexts, which for me have been largely characterized by complex and related operations of privilege. As an anti-racist educator and researcher for the past two decades, my conscious biases may be relatively muted and mitigated in comparison with many others in my social location nexus; however, this is not a productive juxtaposition. I am always working at becoming—becoming anti-racist and becoming productive in the struggle for racial justice. Many of the teachers in this study, while not known to me personally before the project, were by no means strangers. Their whiteness is powerfully familiar and recognizable to me. I locate myself in this work, its analyses, and its political call for anti-racist work in education.

The first section of this article introduces the relevant literature on URB in education. The second section identifies the key theoretical engagements and approaches of the work. The third section offers a detailed description of the study, its methods, and its methodology. The article then turns to the findings, in two distinct but related areas: nonclassroom impacts, which explores teachers’ reflection on the evolution of their thinking and epistemic engagements in their personal and professional lives, and classroom impacts, which explores teachers’ reflections on changes to professional practice with students. This is followed by discussion and implications, as well as a brief conclusion.

## Unconscious Racial Bias: An Introduction to the Literature in Education

URB includes both positive and negative associations with people or characteristics of a particular race—for example, associating qualities such as intelligence, goodness, or threat with people of certain races. Staats et al. (2017) defined unconscious bias as “the attitudes or stereotypes that affect our understanding, actions, and decisions in an unconscious manner. Activated involuntarily, without awareness or intentional control” (p. 10). Staats (2016) argued that unconscious biases “can have a tremendous impact on decision making” (p. 30). Choudhury (2015) explained that although everyone has unconscious biases, “the negative effects of bias are linked to social power and group status in society” (p. 57) and are also linked to behavior within those social power relations. People with less power in terms of gender, race, ethnicity, sexuality, income, neurotypicality, and other elements of social location may experience negative effects of unconscious bias, whereas those with more social power in these domains may be privileged by those same biases.

Ghoshal et al. (2013) investigated successful practices for teaching about URB using the Harvard Implicit Association Tests. Adams et al. (2014) built on this work with a focus on educators. Markova et al. (2016) used a combination of social cognition approaches to illustrate the URB of preservice teachers toward students in special education pathways, while Clark and Zygmunt (2014) documented the challenges of confronting unconscious bias with preservice teachers and the implications for teacher educators.

In their study on postsecondary instruction and evaluation, Jacoby-Senghor et al. (2016) argued that the academic underperformance of racially marginalized students may be impacted by the URBs affecting educators’ pedagogical effectiveness. Kahneman (2011) examined unconscious bias and educator practice, and charted the related impacts on disciplinary practices.

Research suggests that teaching in racially marginalized communities may in some cases reduce URB and unconscious skin tone bias (Dobbie & Fryer, 2015; Mo & Conn, 2018). However, the work of Starck et al. (2020) illustrated the ways in which teachers’ unconscious (and conscious) biases do not significantly differ from racial biases across the general population in the United States and points to significant patterns of pro-white conscious and URB among teachers. The Perception Institute (2014) has determined that “a significant majority of Whites as well as Asian Americans and Latinos show anti-Black [unconscious] bias . . . and almost half of African Americans also show anti-Black bias” (p. 8).

Unconscious bias is more likely to affect our judgments and behaviors when we encounter vague or incomplete information, the presence of time constraints, and circumstances in which our cognitive control may not be optimal—such as being fatigued, suffering from stress, and/or having a lot on our minds (Staats, 2016). The impacts of unconscious bias grow more powerfully determinant in our decision-making during busy, chaotic, rushed, and messy encounters—qualities of daily professional life recognizable to many teachers. Teachers’ conscious and unconscious expectations of students can impact teacher decision-making, students’ academic trajectories, and students’ expectations of self (see Smith et al., 1999; Staats, 2016; Vavrus & Cole, 2002).

In one of the few critical studies addressing URB in education in Canada, the work of Parekh et al. (2018) explored Ontario teachers’ perceptions of students’ learning skills, finding anti-Black URB in teachers’ evaluation of student learning skills (Parekh et al., 2018). Because a large proportion of teachers in Ontario are White, these authors suggested that it is possible that some teachers see students like themselves as more likely to achieve (Parekh et al., 2018).

## Theoretical Frames: Critical Anti-Racist Approaches

While URB is increasingly well understood by social psychologists, there has been limited engagement from critical scholars working in areas such as critical race theory (CRT), anti-colonialism, and critical whiteness studies—despite the popularity of interrogating URB as an anti-racism strategy in education.

CRT in education has laid bare and problematized the central function of schooling in the safeguarding and management of white supremacy (see founding scholars Dixson & Rousseau, 2005; Ladson-Billings, 1998; Leonardo, 2009; Lynn & Parker, 2006; and Tate, 1997; as well as more recent contributions that have extended and expanded these analyses: Kohli & Solórzano, 2012; Ledesma & Calderón, 2015; Leonardo, 2013; and Parker & Gillborn, 2020).

Decolonial and Indigenous scholars complement anti-racist work to consider the colonial operations of race and white supremacy (see Battiste, 2013; Brayboy & Lomawaima, 2018; St. Denis, 2017; Tuck & Gaztambide-Fernández, 2013; Tuck & Yang, 2012). The anti-colonial works of Fanon (1986), Memmi (1965), and others offer a phenomenology of race, which Ahmed (2007) expanded to analyses of whiteness. Drawing on decolonial and anti-colonial approaches, Ahmed suggested that “whiteness is lived as a background to experience” and argued that whiteness is not “an ontological given,” but is instead something that “has been received, or become given, over time . . . an ongoing and unfinished history, which orientates bodies in specific directions, affecting how [White people] ‘take up’ space” (p. 150). This reading is useful for understanding the embodied life and energies of whiteness, racism, coloniality, power, and oppression and the ways in which these socially constructed and socially mediated phenomena are lived by teachers in schools.

An offshoot of critical CRT, critical whiteness studies considers the workings of race and white supremacy at the conscious and unconscious levels, highlighting advantage (or privilege) experienced by White people in specific relation to disadvantage (or punishment) experienced by Black, Brown, Indigenous, and People of Color (BBIPOC) who are socially located outside of whiteness (see Frankenberg, 2004; Gaine, 2000; Gallagher, 2000; Twine, 2004; Wise, 2002). Harris’s (1993) *Whiteness as Property* offers a powerfully grounded formulation of whiteness as material, in both figurative and literal terms, explaining, “Property rights are contingent on, intertwined with, and conflated with race,” an operation through which social relations evolve “to reproduce subordination in the present” (p. 1714). Anticipating Ahmed’s (2007) phenomenology of whiteness, Harris carefully considered the related and mutually reliant operations of unconscious and conscious anti-Black racism, illustrating how systemic and discursive racism produce whiteness as protected property on one hand, while contracting the rights and opportunities for African Americans on the other.

Whiteness is distinct from the color of our skin insofar as whiteness exists in the absence of visible white skin. Consider

someone or something sounding white, acting white . . . or white curriculum, white food, white taste and white culture. While all of these are imperfect categories, they are clear signifiers which most of us can understand and imagine quite easily. (Kempf, 2019, p. 385)

To understand the operations of whiteness at the institutional and structural levels, Morrison (1993) and Thandeka (1999) offer critical insights into the ways in which race, racism, whiteness, and related operations of power and punishment rely on binary social imaginaries. Specifically, whiteness exists, and can only exist, in relation to an imagined non-whiteness. Whiteness is then both visible and invisible, implicit and explicit.

As Juárez (2013) noted, white supremacy is nonetheless a white people problem, and the responsibility for addressing it falls first and foremost to white people. In examining anti-racist research and activism, Leonardo (2013) warned that a failure to name and engage whiteness is unacceptable in the struggle for racial justice. He argued,

It is problematic to focus solely on the margins, which negates a critical look at the center and reinforces the invisibility of whiteness, including Whites’ racial investments and general process of racialization in schools. Once again, they escape critical scrutiny, historical accountability, and moral culpability. (p. 33)

Second-wave White teacher identity studies (e.g., Lensmire et al., 2013) build on CRT, as well as critical whiteness studies, to offer a complicated and critical study of “the cultural production of race, whiteness, and White teacher identities that articulates complex historical and social forces along with related understandings of teaching and learning in context” (Jupp et al., 2016, p.1163).

Among the crucial elements common to all these approaches is the assertion that operations of race and racism must be considered in all aspects of educational life. The texts that make up the selected literature used in this study each offer powerful and interwoven analyses of the historical, institutional, and systemic operations of race and racism.

## The Study, Methods, and Methodology: Toward Deep Work

This project emerged from a dual recognition of URB as a productive entry point for racial awareness and anti-racism work, alongside a significant concern about the failure of mainstream URB discourse to address structural racism and white supremacy—masking at times the deeper ways in which Euro-colonial racism underpins social relations in contemporary U.S., Canadian, European, and other contexts. This work seeks to address these limitations in the design of the study through deep work with participants. Specifically, the study sought to better understand (1) teachers’ professional and personal experiences of racial bias work; (2) the impacts of reading critical texts focusing on systemic, structural, and institutional racism on teacher understandings of their own racial biases; and (3) teachers’ perspectives on the impacts of reading critical texts in terms of their professional practices.

As a White scholar conducting anti-racist work and research, I work to stay aware of the epistemic limitations and possibilities of my social locations, and I am conscious of the real perils of White researchers taking up limited space, time, and resources in anti-oppression scholarship. Consideration is required of what work is appropriate, how and where my privilege is best spent, and how I might work toward racial justice in education.

While the storytelling and counter-storytelling of those targeted by racism (Solórzano & Yosso, 2001) are central to making fulsome meaning of race and racism, I avoid work as lead researcher on projects that center on the collection and analysis of the race(d) testimony of Black, Indigenous, Latinx, South Asian, and East Asian folks. This raced ethic of practice recognizes the oppressive practices (past and present) of white pageantries of racialized pain as disembodied academic object, of the commodification of racialized pain for white gain, of long-standing practices of whitesplaining race in the academy, and of the high costs and harms caused by calls for folks of color to tell their race stories to White folks (Bonilla-Silva & Zuberi, 2008).

### Duration, Participants, and Data Sources

This mixed method study took place over 10 months (one school year) with 12 secondary school teachers, in Toronto, Ontario, Canada. Of the 12 participants, nine identified as White, one as East Asian, and two as mixed race and White-presenting. Three identified as male, and nine as female. All identified as cisgender. Teaching experience varied from two to 22 years. All participants taught at racially diverse schools in an urban center in Southern Ontario. Teachers were recruited at a central, board-run staff meeting with teachers from nine different schools. I made a short presentation in which I described the entire research design and indicated this would be an opportunity to learn about race and racism and reflect on their own racial biases in teaching. All teachers expressing an interest were invited to participate in the study. The study stretched across an entire school year. It was time consuming and emotionally challenging for participants, indicating an exceptional commitment to anti-racist professional learning.

The project was designed using multiple data sources from participants, including electronic survey responses, ongoing journaling/reflection, a midpoint check-in questionnaire, and a final interview. These multiple entry points, as well as the duration of the project, aimed to get at Yin’s (2014) notion of “rival explanations” to go beyond the taken-for-granted and toward deeper understanding over time.

### Phase 1

In the first phase of the research (September to December 2018), participants read selections from *State of the Science* (Staats et al., 2017), an introductory review of key concepts and research on URB. They then completed four online modules introducing URB, offered by Ohio State University, and completed multiple implicit association tests on Project Implicit’s website. Phase 1 culminated in participants attending a presentation given by the research team, during which the team provided a detailed overview of 10 different URB mitigation intervention strategies that have been developed and used successfully in social psychology (Staats et al., 2017; Yale Center for Teaching and Learning, 2018).

The 10 formal interventions were organized into three groups adapted from the Yale Center for Teaching and Learning (2018) and Staats et al. (2017). Group A included professional practices, Group B included interpersonal interventions, and Group C) included personal development interventions. Participants were asked to choose one intervention from each of these categories. In addition to the formal racial bias mitigation activities in Groups A, B, and C, teachers chose one book from the selected literature, on which they would offer midpoint and postreading reflections. The books, in various ways, focus on institutional, systemic, and structural operations of race and racism, engaging CRT, anti-colonialism, and critical whiteness perspectives, reflective of the vital critical perspectives introduced earlier.

Participants completed an online survey in which they selected their intervention strategies and their book, and provided demographic data, brief reflections on the activities completed to date, and brief reflections on race and practice. Teachers worked with research team members to plot and plan the implementation of their chosen interventions to be completed over the next academic term (February to June, 2019).

### Phase 2

In Phase 2 of the research (February to June 2019), teachers shared their ongoing reflections on the interventions and their implementation activities through online and handwritten journaling, phone interviews, and email exchanges. Each teacher then participated in a culminating interview in the spring/summer. Participants were asked to reflect on resonances and dissonances between themes from their chosen book(s) and the interventions from Groups A, B, and C, as well as on any ways in which they connected themes of the book to their personal and professional lives. Final interview questions about participants’ selected books were individualized and developed based on previously submitted individual written reflections.

### Data Analysis

This work sought to anticipate and consider the presence of multiple and contradictory understandings of race, responsibility, and professional practice among participants (Clandinin & Connelly, 2000; Lincoln & Guba, 1985). From the earliest stages, the team was guided by anti-racist education principles as a lens through which to undertake analytical work: for example, a recognition that racism is reproduced in schools, that race and racism should be considered in all aspects of educational life, and that race operates phenomenologically with powerful links between the personal, institutional, and structural domains. We used NVivo software to organize each participant’s survey replies, written reflections, questionnaire responses, email correspondences, and interview transcripts. Transcription was completed by research team members and by a professional transcription company (all by ear). Guided by our theoretical framework, research questions, and extant literature, research team members worked individually on all participant files, making notes on possible inferential and descriptive codes (Creswell, 1998), and memoing (Miles & Huberman, 1994) on key ideas and patterns. The team then came together to share and note these findings, identifying resonances and dissonances among the individual notes across all participant data.

This process resulted in discrete sketches of participants (Creswell, 1998; Merriam, 1998), which we thereafter treated as stand-alone cases (Merriam, 1998; Sandelowski, 1996). Building inductively on this work, the team then began cross-case analysis (Khan VanWynsberghe, 2008) in three broad streams within the data: (1) participant experiences of the social psychology approaches in general, (2) participant experiences of one particular intervention, and (3) participant engagement with and reflection on the selected literature. Discrete thematic analysis proceeded in each of these three areas.

This article focuses on the third stream of analysis. We used a narrative approach to investigate and examine teacher experiences and reflections on the selected literature (see Barone, 2000; Connelly & Clandinin, 1990; Moen, 2006). Under the largely descriptive categories of classroom and nonclassroom effects, the team drew on earlier coding and memoing work (see Appendix for a sample of inductive themes and inferential codes) to identify warranted analytical foci in cross-case perspective, which are reflected in the findings that follow.

### Reliability and Limitations

The research team engaged a variety of common qualitative research techniques for bolstering the trustworthiness of the project’s findings (Creswell, 2009). Each team member paid close attention to, and memoed about, our experiences within the work as socially located actors. As early as the beginning of Phase 1, we noted a series of disparities in our experiences, which we agreed felt race- and gender based. As a middle-aged White man and former high school teacher in Toronto, I repeatedly experienced White participants and their reflections differently than did both of the two research assistants, who were younger women of color and had not been teachers. We used these discrepancies productively, first ensuring the social safety of the research assistants, and then working explicitly through the implications for testing and strengthening our respective interpretations. We performed simultaneous preliminary explorations of multiple data sources (reflections and interviews). This functioned as a form of raced and gendered triangulation, targeting invalidating data, in service of truthful, accurate, and rich portraits of participants’ reflections and experiences.

During both phases of data collection, and throughout the data analysis process, research team members wrote and shared analytic memos (Miles & Huberman, 1994) about specific data sources and ideas (e.g., teacher resistance to specific ideas in the literature). We also met regularly to share and compare preliminary observations, concerns, and codes. We co-reviewed relevant theoretical literature to orient our thinking. As part of an effort to generate reliable codes and a reliable coding process, the team engaged in an iterative open coding process, first looking at each participant one at a time, as described earlier, and then developing cross-case analyses in the three streams identified in the preceding section. We paid careful attention to discrepancies in the codes we each assigned, revising accordingly, with the goal of agreement and clear cross-team understandings (Creswell, 2009).

The research presents a series of limitations. First, the study is limited to an urban center in Ontario. Second, the sample was limited to the teachers who chose to participate, so caution is in order because a self-selection filter (as with much research) exercised by the subjects themselves sets in motion the biases of interest level, comfort level, etc. Third, the sample size is not representative. Fourth, as with social research contained by a given time frame (10 months), findings must be carefully contextualized not only in terms of place, but also in terms of time—a matter that bears somewhat uniquely on this research in the context of global movements for racial justice. Broadly, restraint is in order here against generalizability.

Additionally, this article does not attempt to provide empirical evidence for the efficacy of URB mitigation techniques. In narrow focus, this work looks at teacher reflections on the critical nonfiction texts, with a focus on related implications for professional and personal engagements with racism and white supremacy. These core limitations notwithstanding, this work raises important questions regarding anti-racism work in Canadian education, potentially generating new questions applicable to teachers elsewhere.

## Findings: Participant Engagement With the Selected Literature

In an effort to interweave critical anti-racist approaches with work from social psychology, all participants read at least one book from the selected literature. This section presents findings from these engagements in two areas: nonclassroom effects and classroom effects.

### Nonclassroom Effects

Notable nonclassroom effects are organized into three overlapping areas: (1) participants seeing race differently, (2) participants interrogating their own racial autobiographies, and (3) participants speaking up against racism.

#### Nonclassroom Effect 1: Seeing Race Differently

In terms of seeing race differently, teachers reported noticing the operations of racism and white supremacy differently and in more places. Specifically, teachers reported a shift in how and how often they were noticing race. The selected literature pushed some teachers to think more deeply about the experiences of BBIPOC. Laura, a midcareer White teacher, began thinking more deeply about the daily race work required for many BBIPOC, explaining, “Reading *White Fragility* [by Robin DiAngelo, 2018], I am reminded very quickly that people who are not White are often . . . trying to decode, feeling like the ‘other,’ and ultimately being marginalized.”

Veteran White teacher Jennifer explained that the most profound impact of reading *Policing Black Lives* by Robin Maynard (2017) was in how she came to view her Black colleagues. She wrote,

I have more understanding of the possible struggles and perceptions from others that they may have encountered, and recognize more and more my privilege. I feel that I am more aware of their blackness and my whiteness. . . . Most importantly, I am listening, observing and learning and reflecting a lot more than I ever did. As well, I have more compassion and empathy for my son and his father.

Jennifer’s recognition of blackness as a lived experience characterized too often by oppression offers a reflexive understanding of the ways in which her own racialization as White is characterized by privilege and advantage. Powerfully, the reflection ends with Jennifer having more compassion for her racialized family—pushing back on the myth that White folks’ proximity (whether through family or friends) to BBIPOC folks negates the need for their own critical race work and understanding.

#### Nonclassroom Effect 2: Reflexive Racial Autobiography

The second nonclassroom effect centered on reflexive racial autobiography. Engaging the selected literature helped many participants interrogate their own past racial engagements, leading to reconsiderations of the way race and racism had impacted their past experiences and decisions.

While reading and reflecting on *Policing Black Lives*, Tanya, a veteran mixed-race, White-presenting teacher, reconsidered an interaction from years ago, explaining,

When I read about White landowners refusing to rent to Blacks, resulting in Black communities being restricted to segregated housing, I had to question my own biases. I have a house that I rent out. . . . A woman who consulted for a First Nations Health Centre wanted to rent it. I denied her application. My rationale was that she did not have a salaried income. However, I was aware, but kept that awareness far from the surface, that I was influenced by the fact that it was a First Nations institution funding her. My bias as to the security of such funding was definitely in play.

Shawna, a veteran White teacher, also read *Policing Black Lives*. She was driven to entirely rethink the way she had understood police and policing, as well as the way she had taught her own children to regard police officers. She explained,

I think one of the biggest learnings I had was around the history of the police. . . . [my Black colleague] is severely phobic of the police and she had obviously been traumatized by them and had extremely bad interactions with them. . . . My entire experience as a White female has just been one of like friendly protection and for years, I’ve said to my children, we’re on the good guy team, who else is on the good guy team? The police and the firefighters and the nurses and there’s a very strong sense of [the] good guy team.

Shawna was connecting race and policing to her most intimate professional and personal experiences and engagements. Shocked that her understanding of the “good guy team” was not universal, she had more information, and thus empathy for her colleague, as well as a new understanding of the exceptional nature of her relationship to police—one characterized by friendly protection.

#### Nonclassroom Effect 3: Speaking Out, Speaking Up

The final nonclassroom effect centered on teachers speaking out against racism when they previously might have stayed silent. This effect included conversations with family members, colleagues, and strangers. Most notably, Laura (quoted earlier) offered a variety of anecdotes from the year of the study, detailing the ways she had spoken up and out against racist behavior. One incident included a confrontation on the metro with a group of young skinheads, which almost turned violent. Another detailed an embarrassing public moment with a family member. A third described a difficult conversation in a crafting class. Her story that follows centers on this last instance:

Everyone in the class was White, including the instructor, save one person. Working away, someone started talking about a recent trip to Black Creek Pioneer Village.^2^ They were going on about what a great time they had and proceeded to talk about how idyllic it would have been to live in [the 19th century], until I interrupted with “unless you were Black.” The conversation stopped and the room stayed pretty silent until someone started talking about something else. . . I don’t share these as examples of my righteousness. But they are what are for me knee-jerk reactions that help to “break the pact.”

Laura concluded with a reference to breaking the pact of white solidarity, as identified in *White Fragility* as an anti-racist practice for disrupting white supremacy at the discursive level. Opportunities for disruption, such as the one Laura described, are familiar to most people. Few White folks, however, regularly speak up to breach whiteness in the way Laura described. The act was neither revolutionary nor unique; however, it is uncommon and important. Uninterrupted, White folks’ comfortably waxing nostalgic (waxing ignorant in the way described) can reproduce white supremacy.

While Laura’s stories of speaking up concerned various personal situations, Rachel, a White midcareer teacher, reflected on her engagements with Paulette Regan’s (2014) *Unsettling the Settler Within* by sharing a powerful story about an exchange with a White colleague at school, included here at length:

After speaking openly with all my colleagues about [a piece of art] I had seen the night before made by a Cree [artist] about a tragedy in her community . . . [my colleague]—as [an artist] herself—expressed anger that funding was not available to her. . . . According to her, funding can now only be accessed by “Indigenous people and women of colour.” She went on to tell my [sic] how this was an example of “political correctness” gone wrong.

I reiterated it was an important story told by one of the few people who could and should tell it. I then drew her attention to the word “correctness” and said that I actually thought that it was indeed a correction of a historic wrong—a reparation in fact—and that the inclusion and promotion of Indigenous voices in the arts was critical as we move forward in our efforts of reconciliation . . . I was glad to have felt brave enough to challenge her complaint and discuss it openly.

Such misunderstandings about race and resources (whether arts funding, vaccine distribution, education priorities, etc.) are common fare on the menu of many white-on-white conversations. Rachel, informed by the selected literature, was empowered to push back against this racist trope. She felt not only comfortable doing so but also glad she had done it after the fact.

The nonclassroom effects—participants seeing race differently, interrogating racial autobiographies, and speaking up—sketch a variety of ways in which the selected literature impacted participants. While these center on engagements with racism and white supremacy in various contexts, the next section focuses specifically on schools and classrooms.

### Classroom Effects

Classroom effects are notable in four areas: (1) developing a new sense of responsibility, (2) building excitement and emotional capacity for authentic engagement with race and anti-racism, (3) critically reflecting on past practice, and (4) making plans for future professional practice. Each focuses on changes to understanding, and operationalizing race-informed teaching practices.

#### Classroom Effect 1: A Sense of Responsibility

Broadly, engagement with the selected literature led some participants to rethink their racial responsibilities as teachers. Veteran White teacher Meghan shared a story about a presentation from the school board on its decision to phase out student streaming based on perceived academic suitability. The presentation highlighted the overrepresentation of Black students in nonacademic pathways. Meghan recalled that other teachers in the presentation began to raise objections. She explained, “These teachers were just complaining . . . we can’t do this.” This struck an off chord with Meghan, and she reflected on how she was pushed in a different direction than her colleagues:

When we look at this data, like, how can we not have a moral obligation to try and work on [addressing this]? Absolutely, this will be challenging for a little while, but when we are presented with this data, we can’t in good conscience, say, “No, we need to keep things as they are.”

The push to eliminate academic streams in Ontario, known as de-streaming, is a hot-button issue among teachers, with opposition from many who feel that there must be accompanying supports, which they reasonably fear will not be provided. Meghan was aware of these concerns and yet felt compelled by the data. *White Privilege* has given her “a new sense of responsibility,” which has her feeling, in her words, “reinvigorated. . . . I have started thinking more concretely about this kind of stuff again, which is good . . . [the book] brought a lot of excitement to the surface.”

Meghan connected the reading to both a new enthusiasm and a new commitment to the heavy lifting of inclusive de-streaming, even in the likely absence of sufficient supports. While the responsibility for racial justice work cannot rely on individual teachers, it most certainly cannot proceed absent the anti-racist commitments of teachers.

Paul, an early-career White teacher, also read *White Fragility*. He was one of two participants who took issue with the content of the readings, raising concerns about the qualifications of the author. Nonetheless, when asked about the implications of the book on his work and thinking, he was clearly impacted, offering,

I think that there were definitely important lessons . . . in terms of remembering this is my privilege . . . I have a society where I benefitted to get into this position. I had things working for me that students who are say, Black, might not have had. So, if I do move up into a position where I feel like I can make active change at the policy level, I would probably rely on some of the messages from this book, and the fact that I have to consider the history of racism.

Notably, for Paul, there was no apparent application to immediate practice; however, he expressed a new understanding regarding his own place in education and the role of history in contemporary race power. Like Meghan, he identified a feeling of obligation in terms of practice after engaging the selected literature.

#### Classroom Effect 2: Developing Excitement and Emotional Capacity

Teacher engagement with race and racism in education can be slow, hard to see, hard to track, and hard to quantify. The selected literature seems to have supported teachers in building the emotional capacity for authentic engagement with race and anti-racism in teaching and in generating excitement for this work.

Jennifer’s comments on *Policing Black Lives* echo Meghan’s enthusiasm noted earlier. She explained, “For the first time in my life, I am actually interested in the social studies curriculum. Up until now I have always avoided teaching it by trading it off for Science and Technology with another teacher.” Thus, the selected literature has led to deeper engagement with the existing curriculum for Jennifer.

Ralph, reading *Unsettling the Settler Within*, described a similar enthusiasm developing from the engagement with the reading. He said,

[The book] makes me feel excited for that work, and helps me understand . . . and navigate the emotional responses that a lot of people are going to have to that. Because I think it is very foundation-shaking to start to look at Canadian colonialism.

Ralph is preparing himself for the discomfort and challenges awaiting anyone doing critical work in mainstream spaces—for the “foundation-shaking” this work and its implications will cause in classrooms and perhaps in terms of his own footing therein.

Other teachers, reflecting on the selected literature, remarked on the need to get out of their own way—the need to move past ego, guilt, and self-consciousness to focus instead on authentic attention to race and racism in service of BBIPOC students and their communities.

Sarah was struck by the push in *White Fragility* for White people to move from a focus on guilt to a focus on building awareness. She wrote,

When DiAngelo expresses her thoughts on guilt, it resonates with me because I feel the exact same way. DiAngelo writes, “I am sometimes asked whether my work reinforces and takes advantage of white guilt. But I don’t see my efforts to uncover how race shapes my life as a matter of guilt. I know that because I was socialized as white in a racism-based society. . . ” [from DiAngelo, 2018, p. 149]. As an [educator] and a White woman, I don’t believe that this will change. I continuously attempt to not feel guilty but rather increase my awareness of race and its impact on youth.

Sarah’s thinking resonates with reflections from Laura, who described how she felt while at a dinner she attended as part of the research project. There, she and other White teachers listened to Black and Brown parents and students describe their experiences of race in schools. Drawing on *White Fragility*, Laura explained,

I worried a lot about . . . how I would be perceived by the guests, my colleagues, and the study staff. . . Could I not find the words because I am ultimately unprepared to have this conversation? In this moment. . . White Fragility really resonated . . . I don’t know that my preoccupation was exclusively about not being perceived as racist (because I do acknowledge that I am racist—certainly reading Robin DiAngelo’s book has given me a vocabulary to talk about how and why), it was more a concern that I be seen as someone who is engaging meaningfully with this process, as a person who wants to “bear witness to the pain of racism that we cause” [DiAngelo, 2018, p. 128].

Laura credited *White Fragility* with giving her the vocabulary to discuss her own racism, and, importantly, the tools with which to work through her feelings in the process—feelings that so often shut down white engagement with anti-racism. She went on to interrogate this further, writing, “I want to be seen as one of the good guys and I fully appreciate that desire keeps it about me, and that is NOT the point.” She is referring here to DiAngelo’s (2018) caution against White folks feeling that being positively “perceived by others is the most important issue” (p. 121). Laura explained,

I see that upon first reading I had put a huge star next to this line and wrote in the margin “it’s not about me!” So I get that I got stuck here, in my own preoccupation with my self-image. This is clearly an area I need to focus in on.

This important shift illustrates a navigation away from solipsistic centering of whiteness (and white comfort) to an approach that acknowledges the racial damage caused by White people and white supremacy in society in general, and through formal schooling in particular.

#### Classroom Effect 3: Critically Reflecting on Past Practice With Students

The selected literature prompted teachers to reflect critically on past encounters, thoughts, and actions in their professional practice generally, as well as with specific students and groups of students. Reflections vary, from noting misunderstandings about student perspectives and experiences, to reconsiderations of race language use.

Jennifer, who read *Policing Black Lives*, reflected on the way she had previously struggled to fully comprehend reports from BBIPOC students about racist police practices. She explained,

My students tell me stories. It’s undeniable that Black people are targeted. Many years ago, there were numerous shootings in Regent Park^3^ resulting in death, that went unreported. This confused me and at times I doubted my students [were telling the truth about these unreported deaths of Black people. Nowadays [the media] are reporting the shootings regularly but I am inferring this is because the shots are not restricted to the Black neighborhoods but have entered into the playgrounds of the White communities.

*Policing Black Lives*, which details experiences of violence and policing in African Canadian communities, offered Jennifer a deeper understanding of what she had previously been unable to hear from her students.

Laura’s experience was similar to Jennifer’s, and after describing her own positive relationship with police, Jennifer explained the ways that her understanding has evolved:

Reading the book gave me the understanding of . . . why the language of my [students], their experience of when I bring the police in the building, how frightening that is on an elemental level that they don’t see these as people who protect them. They see them as people who actively hurt them.

Police are regularly called to Laura’s school, so this realization is crucial for understanding student experience, and thus key for supporting learning and well-being.

Sarah was pushed by her reading of *White Fragility* to reconsider her own choices of terminology. She explained,

DiAngelo’s theories are spot on. I tend to avoid seeing or reflecting on my students of colour. . . . The term “aversive racism” was new for me because previously, I couldn’t put a label on my past behaviours. For instance, to “maintain a positive self-image [while] avoiding direct racial language and using racially coded terms such as urban. . . ” [p. 43]. I was not fully aware that [as a result of such acts] “people of colour are blocked by moving forward” [p. 50]. I believe that my intent is genuine but I was unaware of the impact . . . I see race and race sees me.

Seeking her own behavior in *White Fragility*, Sarah concluded that she now sees race (and is seen by race). Although she already felt committed to equitable and inclusive practices, this profound shift represented a turn away from color-blind(ing) pedagogy, which she had previously thought was the right way to approach race in education.

#### Classroom Effect 4: Implications for Future Professional Practice

Teachers felt that reading these books would inform their teaching going forward. Some teachers had specific practices in mind, including attention to course planning, course content, and special events, while others were more general and philosophical about their aims and plans.

When asked about how engagement with the selected literature might impact her future planning, Laura offered the following on course design:

I’m talking now with my staff around our GLE and GLS courses^4^ next year. Two years ago, when I was talking to staff about that, I was talking like “let’s really increase the literacy skills and let’s work really hard on writing.” Now my language around that is our kids are getting arrested all the time and they’re getting beat up and they’re getting hurt and they’re getting intensely traumatized by that. When we do GLE, GLS this year, can we talk about what is safety for them walking through the streets? What language should they use, how should their bodies be held when they’re getting arrested in order to have them less brutalized?

The GLE and GLS courses are designed to support students who may have special education needs and/or require increased academic supports. Laura’s student population is mostly low-income Black folks. For her, the idea of teaching students how to interact safely with racist and violent police is a meaningful life skill.

Reflecting on *Unsettling the Settler Within*, Shawna, a White midcareer teacher, explained her thinking about a new course:

As I will be teaching [a Grade 9 course titled Expressions of Indigenous Cultures] for the first time this school year, I feel that at least I know a little of where to place myself within the larger context of Native Studies. As a White teacher, I feel I should make my position known. Yes, I am an ally on this learning journey with everyone in the class, as well as Elders and Knowledge Keepers.

Newly, Shawna is centering her identity and position (as a White person in a colonial context) within a settler curriculum that centers on Indigenous culture and people. She is newly thinking through the way she will include and privilege Indigenous voices in her teaching.

Some teachers had already implemented changes to their practice based on their engagement with the selected literature. Rachel, who read *Policing Black Lives*, described her approach, saying, “I have used it to make connections to [the television documentary] *The Skin We’re In*^5^ as part of an assignment for . . . [the course] Society Challenge and Change.”^6^

Jennifer had also already used *Policing Black Lives* to inform her practice. She described her thinking and participation in a Black History Month event as follows:

In the past . . . [i]t seemed to be hard to find Canadian content. I made sure our activities focused mostly on Canadians due to the fact that I realized how unfamiliar I was with many of the Black Canadian references when reading this book. I wanted the students and teachers to see the accomplishments of Black Canadians with the hope that they would make connections and start to see their own trajectory of possible pathways.

Although we might lament the limitations of Black History Month, adequate content information for even the most basic of school assemblies is often missing for many teachers in Canada. When asked if the book inspired any curriculum connections, Jennifer answered, “Most definitely. All I can think about is that I want to reread this book with the goal of developing curriculum for Grade 7 and 8 history.” As a reminder, this is the same participant who was excited for the first time to teach social sciences.

In addition to these specific plans, some participants offered more general reflections. Garth, a veteran mixed-race, White-presenting teacher who read *Monstrous Intimacies* by Christina Sharpe (2010), reflected on the book’s impact on his practice as follows:

I mean we don’t want to turn school into a place where everything is a conversation about what’s wrong with our society, but we do I think want to turn schools into places where we can perpetuate more sound relational dynamics between groups and peoples, and I think that that means we’re addressing our hurt.

Perhaps in pursuit of just the sort of relational dynamics Garth described, when asked about impacts on her practice, Sarah explained,

I believe that my genuine intent to truly support students of colour is a start, and then to build/maintain that trust which is crucial for any kind of positive results. I really appreciated the strategies that DiAngelo [2018] suggests when white fragility surfaces: “breathe, listen, reflect, etc.” [pp. 147–148]. As in terms of next steps, I have to remember that my feelings of guilt, shame, and intimidation are not to interfere with my primary goal—help students move forward.

Similarly, when asked about how Regan’s (2014) *Unsettling the Settler Within* might impact his future practice, Ralph, a midcareer White teacher, reflected,

It was really hitting me hard . . . I was thinking a lot about the history teacher in our school. And like a lot about how history is taught. . . ., about the often-obscured history and relationship (i.e., Canadian colonialism) and considering how to do it while supporting learners to retain hope.

Ralph’s reference to hope resonates with Sarah’s mention of trust, as well as with Garth’s notions of building relational dynamics and addressing pain. Although perhaps liminal aspirations, these are some of the very conditions on which anti-racist practice relies. As such, these ideas form part of the concrete social foundation needed to establish and sustain practices of racially just teaching and learning.

Together, the classroom and nonclassroom effects sketched earlier illustrate the powerful impacts of teacher engagement with critical anti-racist and anti-colonial texts. The next and final section offers a discussion of these findings, with attention to the implications of the work.

## Discussion and Implications

As the classroom and nonclassroom impacts illustrate, teachers’ thinking, engagement, and practices were significantly impacted by the selected literature. Teachers were pushed to rethink racism and white supremacy in the world around them. They reconsidered their racial autobiographies in both the personal and professional realms. Teachers spoke out more about race with family members, strangers, and colleagues. Teachers arrived at new raced understandings of their professional responsibilities, reconsidered past practices with BBIPOC students, and made plans to change and adapt their teaching going forward. They critically reflected on their identities and responsibilities regarding race, colonialism, and history. These findings suggest that URB work may be strengthened and more impactful when strong critical grounding accompanies the proven techniques emerging from social psychology research.

Specifically, many teachers developed a critical reflexivity of accountability, which responds powerfully to Leonardo’s (2013) call for the critical scrutiny, historical accountability, and moral culpability of White folks. This reflexivity emerges phenomenologically and, for many teachers, was an experience of shifting whiteness in which the taken-for-grantedness of whiteness (Ahmed, 2007) and white supremacy became instead named, seen, and accounted for in teaching practice and in their personal lives. Where Ahmed (2007) described whiteness as “lived as a background to experience . . . which orientates bodies in specific directions, affecting how [White people] ‘take up space’” (p. 150), this work brought to the foreground the ways in which participants are oriented by and within Canadian white supremacy in education. The literature proffered a politically implicating background against and into which participants processed and considered their role(s) as educators—their roles as named characters in the historical and contemporary drama of Canadian white supremacy.

Teacher engagements with the selected literature illustrate the deep links between the personal and the professional, suggesting that meaningful growth in only one domain may be difficult, if not impossible, without growth in the other. Can one be a racist neighbor but somehow not a racist teacher? This bleeding of worlds highlights the limitation of professional development for teachers that focuses exclusively on classroom practices and tools. Teacher engagements with the selected literature consistently connected the personal to the institutional and to the systemic. This comprehensive accounting for race allowed teachers to identify daily professional practices as products and producers of racial justice and injustice in schools and society. This work allowed for critical reflection to seep into the most intimate and invisible moments of operationalized whiteness. For example, Laura’s story of disrupting the crafting class, although less dramatic than confronting skinheads on the metro, highlights the often hidden operations of racism and white supremacy at the transhistorical level—at the level of the embraced imaginary of white past.

Laura’s small disruption is perhaps just as important as the subway altercation, if only because skinheads are an obviously irreputable embodiment of whiteness and white supremacy. Skinheads lack subtlety and charm to most onlookers. They make up a reflexively redemptive spectacle for other White folks, forgivingly signaling that the real racists are marked by shaved heads and loud tattoos rather than by quiet and polite commitments to segregated neighborhoods and schools. Long called for by critical race theorists in education (see Kohli & Solórzano, 2012; Ledesma & Calderón, 2015; Leonardo, 2013; Parker & Gillborn, 2020), this race-naming and rupture at the experiential level highlights teacher complicity and pushes for an account of race in the first instances of personal and institutional life.

This work was neither easy nor quick. Rather, it was long, uncomfortable, and, at times, upsetting—the things that conventional URB training tends not to be. The hard, awkward, sometimes painful conversations worked to shift thinking and practice, and the commitments of the participants enabled the deep engagements detailed earlier. These growing pains prepare the emotional terrain for getting out of one’s own way in race work and for cultivating the courage and humility needed for White teachers to work in service of BBIPOC students.

Reading of the selected literature was, methodologically, somewhat out of step with the other elements of participant engagement. However, 10 of the 12 participants noted direct connections between the books and the other types of interventions (professional practices, interpersonal interventions, and personal development interventions). This suggests an important complementarity between teacher intervention practices emerging from social psychology, and the introduction and engagement of critical anti-racist and anti-colonial texts in terms of teachers’ work for racial justice.

In addition to suggesting that teacher experiences of racial bias mitigation are strengthened and deepened by simultaneous and sustained engagement with critical texts, the study suggests that further research on the inverse phenomenon may be called for as well; to wit, might combining professional practices, interpersonal interventions, and personal development interventions (i.e., Groups A, B, and C) with critical reading deepen the impacts of the selected literature? Teacher anti-racist reading groups and teacher professional learning communities are increasingly popular. Such activities may be more impactful if combined with interventions such as those used in this study.

Also falling beyond the scope of this article, this work raises questions about teacher professional engagement with teaching and curriculum. The depth of engagement, excitement, thought, and commitment described in the findings (best exemplified by Jennifer’s new enthusiasm for teaching social studies) may offer insight into teacher engagement and disengagement with curriculum, as well as about the need for a more race-critical curriculum. Without prompting, almost all teachers developed specific and/or general plans for future classroom practice. This reveals a potential avenue through which we may better understand critical engagement of and by teachers on issues of racial justice. Education scholarship on engagement may be overly focused on students, overlooking the way teachers might be energized by opportunities for critical engagement. Recognizing widespread calls for racial justice in education and the persistent difficulties that schools have had in answering these calls, this possibility should be more closely explored.

## Conclusion

This article has argued that reading and reflecting on critical nonfiction literature on racism and white supremacy, in combination with participating in a series of other racial bias mitigation interventions, had a significant impact on the way that participant-teachers see, understand, and address race in their personal and professional lives. This article has not sought to prove or disprove the efficacy of any of the social psychology approaches (intervention Groups A, B, and C), nor to measure changes in teachers’ URB. Instead, this work has sought to better understand teacher engagement with the literature alongside their experiences with other elements of the study.

Findings indicate that reading critical texts alongside racial bias mitigation strategies increased teacher participants’ confidence, ability, and interest in addressing race and racism. Additionally, these engagements helped make connections between individual actions and issues of systemic racism in and out of the classroom, allowing for reflexive self-location by participant-teachers, in service of racial justice in education.

This work offers a detailed look at one approach to critically grounding URB work in education. In contrast to one-off quick sessions on URB, which often focus exclusively on the level of the individual, this work has taken a longer and deeper approach. By making connections between race and racism at the institutional and systemic levels, and the participants’ daily raced experiences, this work offers a way forward for critical approaches to URB work in education.

## Appendix

### Sample of Early Cross-Case Inductive Themes and Inferential Codes

#### Theme: Change

Codes

- Change in practice
- Change in vocabulary
- Self-conscious change
- Awareness of journey in anti-racism work
- Move beyond acknowledgment of privilege

#### Theme: System structure

Codes

- Lack of POC in teaching
- No leadership on race
- Settler-colonialism

#### Theme: Nice

Codes

- Nice White
- Good White person
- Performing race
- Complicating niceness

#### Theme: Privilege

Codes

- White privilege
- Unconscious privilege

#### Theme: Race Talk

Codes

- White–BIPOC race talk
- White–white race talk
- Naming/seeing race
- Naming raced power
- Taboo
- Seeing race
- Naming whiteness
- Taken-for-grantedness
- White gaze
- Self-perception

## Notes

1. Following Harris (1993), this article understands white supremacy as not only “the self-conscious racism of White supremacist hate groups” but also the “political, economic, and cultural system in which Whites overwhelmingly control power and material resources, conscious and unconscious ideas of white superiority and entitlement are widespread, and relations of white dominance and non-white subordination are daily re-enacted across a broad array of institutions and social settings” (p. 1714).

2. Black Creek Pioneer Village is a local tourist attraction that features largely uncritical historical reenactments of settler pioneer life.

3. Regent Park is a low-income mixed-race neighborhood in downtown Toronto.

4. GLE and GLS are two courses designed to offer specialized and targeted academic supports to students.

5. *The Skin We’re In* refers to Desmond Cole’s (2017) television documentary series on race and racism in Canada, not to the 2020 book of the same name.

6. Society, Challenge and Change is an Ontario secondary social science course.
